# The Relationship Between Chinese College Students’ Upward Social Comparison and Involution: Fear of Negative Evaluation as a Mediator and Self-Construal as a Moderator

**DOI:** 10.3390/bs16050624

**Published:** 2026-04-22

**Authors:** Li Dong, Mukaidaisi Maimaiti, Huijia Chen

**Affiliations:** 1 Xinjiang Key Laboratory of Mental Development and Learning Science, Xinjiang Normal University, Urumqi 830017, China; 18040778914@163.com (M.M.); 18160508831@163.com (H.C.); 2 College of Psychology, Xinjiang Normal University, Urumqi 830017, China

**Keywords:** involution, upward social comparison, fear of negative evaluation, self-construal, Chinese college students

## Abstract

With the rapid economic development and social transformation in China, involution has drawn increasing attention across various disciplines. To test the generalizability of previous findings, the present research examined the relationship between upward social comparison and involution, the mediating role of fear of negative evaluation and the moderating role of self-construal in this link. Two studies were conducted. In study 1, 1549 Chinese college students completed four scales comprising Involution, Upward Social Comparison, and Fear of Negative Evaluation. Results indicated that upward social comparison was positively correlated with involution and fear of negative evaluation significantly mediated this relationship. In study 2, 392 college students participated in a priming experiment of self-construal and completed a battery of scales same as Study 1. A significant moderation of self-construal was observed between upward social comparison and fear of negative evaluation. Specifically, for individuals with the independent self-construal, upward social comparison had a weaker impact on fear of negative evaluation. For individuals with the interdependent self-construal, upward social comparison had a stronger impact on fear of negative evaluation. These findings highlight the complex interplay among upward social comparison, fear of negative evaluation, and self-construal in shaping involution among Chinese college students.

## 1. Introduction

In recent years, China has undergone profound and rapid system-wide social changes, which have exposed many young people to involution. As a result, involution has become prevalent among Chinese young people. Involution was first proposed by anthropologist Geertz (1963) when he was explaining the changes in the agricultural structure of Indonesia. It referred to a kind of extensive economic growth model that merely increased input and production scale, rather than pursuing transformation in the mode of production. In recent decades, involution has become a social phenomenon, defined as an adverse psychological state and non-benign competitive behavior when people are in an environment with limited resources and intense pressure (Zhang et al., 2024). In short, involution refers to excessive internal competition without real progress (Huang, 2002) and is closely related to the human need for advancement.

Previous studies have shown that involution can, to some extent, stimulate individuals’ motivation and perseverance (Niu, 2020; Ling & Li, 2022). However, involution can also impair physical and mental health of individuals, inhibit innovation, cause waste of resources and lead to a vicious cycle of competing for the sake of competing (Zhan et al., 2023; Zhang et al., 2024).

With the transformation and development of Chinese society, involution initially emerged online (Y. Chen & Xie, 2023) and aroused hot discussions among young people seeking opportunities for further education, employment and career promotion. Involution reflects a relatively stable attitude and the social behaviors of these young people in pursuit of self-development. Specifically, Chinese college students are in a critical period of self-awareness and value formation, while facing dual pressures of academic performance and employment (Lin, 2021; Qin & Dai, 2022). Therefore, the characteristics and the influencing factors of involution among Chinese college students have become important issues of concern in contemporary China.

## 2. Literature Review and Theoretical Hypothesis

### 2.1. Upward Social Comparison and Involution

In a highly competitive society, involution is essentially driven by individuals’ need to maintain their social image and status (Liu & Ru, 2022), and this need can be easily stimulated and strengthened through social comparison. Social comparison theory proposes that individuals tend to evaluate themselves by comparing with others when objective evaluation standards are absent (Festinger, 1954; Gerber et al., 2018). Upward social comparison occurs when individuals compare themselves with others who possess more positive or superior characteristics (Wills, 1981; Xing & Yu, 2005). As a type of social comparison, upward social comparison can affect an individual’s self-perception and goal setting, and intensify the sense of threat, which ultimately leads to competitive behaviors (Appel et al., 2016; Nesi et al., 2018). Upward social comparison has become an important driving force for individuals to engage in involution. In China, intensified competition and scarce high-quality resources directly fuel the anxiety driven by upward social comparison—namely, the fear of losing one’s relative standing. This anxiety compels individuals to exert excessive efforts to close the perceived gap, thereby perpetuating a cycle of involution (Ma & Huang, 2021; J. Wang, 2021). Therefore, based on the findings and the theoretical view mentioned above, we propose Hypothesis 1:

**H1.**  *Upward social comparison is positively associated with involution among Chinese college students.*

### 2.2. Upward Social Comparison, Fear of Negative Evaluation and Involution

To better understand how upward social comparison influences involution, it is necessary to further analyze the underlying mechanisms of this relationship. When competition has permeated various aspects of contemporary life, one of the adaptive mechanisms for human beings is the avoidance of failure (Fan & Zhang, 2024; Jiang et al., 2024). Specifically, people develop a relatively sensitive and avoidant tendency toward any potential negative evaluations. Watson and Friend (1969) defined fear of negative evaluation as apprehension about others’ evaluations, distress at their negative evaluations, avoidance of evaluative situations, and the expectation that others will evaluate oneself negatively. In short, it refers to an individual’s concern and fear of receiving negative appraisals from others. It has been acknowledged that fear of negative evaluation is adaptive for human development because it can help people avoid the risk of social exclusion and maintain their opportunities for survival and reproduction (Gilbert, 2001).

However, fear of negative evaluation may induce anxiety, avoidance, and feelings of inferiority. Studies have shown that upward social comparison, as a source of pressure, can make individuals doubt their abilities, experience a sense of psychological crisis, and therefore intensify their fear of negative evaluations (Adelman & Dasgupta, 2018; Weeks & Howell, 2012). In order to avoid being labeled as “lagging behind” and reduce the risk of negative evaluation, Chinese college students have to exert considerable efforts to meet performance-oriented standards (Yang et al., 2022; T. Zhou & Miao, 2023) and construct a “competent” or “excellent” image on campus (Qin & Dai, 2022; T. Zhou & Miao, 2023).

Previous studies have found that social comparison influences fear of negative evaluation, and fear of negative evaluation is likely to induce stress among college students (Nonterah et al., 2015; Pigart et al., 2024). Moreover, Li et al. (2026) insisted that upward social comparison makes Chinese college students perceive the gap clearly, thereby generating fear of negative evaluation, which in turn leads to feelings of pressure. Fear of negative evaluation plays a mediating role between upward social comparison and pressure.

Based on the above analysis, fear of negative evaluation is not only a psychological response induced by upward social comparison, but also a factor that aggravates involution. Therefore, Hypothesis 2 is postulated:

**H2.**  *Fear of negative evaluation plays a mediating role in the relationship between upward social comparison and involution.*

### 2.3. Upward Social Comparison, Fear of Negative Evaluation, Self-Construal and Involution

J. Chen et al. (2024) affirmed that not all anxiety or fear of negative evaluation caused by upward social comparison leads to involution. This suggests that there might be individual differences in the relationships among upward social comparison, fear of negative evaluation and involution among Chinese college students.

Originating initially from cross-culture comparative studies, self-construal refers to how individuals view the relationship between themselves and others (Markus & Kitayama, 1991). It can be divided into independent self-construal and interdependent self-construal. Independent self-construal focuses on personal autonomy and uniqueness. People with independent self-construal tend to view the self as an independent entity. Interdependent self-construal focuses on the relationship between the self and those around them. People with interdependent self-construal tend to view the self as an interdependent entity.

Social comparison is important for individuals to evaluate themselves and develop their self-concept. Previous studies have explored the moderating role of self-construal in the differential effects of social comparison on individuals’ self-evaluation (Cheng & Lam, 2007; Stapel & Koomen, 2001). When independent self-construal is activated, social comparison tends to make an individual’s self-evaluation deviate from the comparison target. When interdependent self-construal is activated, social comparison tends to make an individual’s self-evaluation shift toward the comparison target. Therefore, these results indicate that independent self-construal and interdependent self-construal play different roles in shaping self-concept resulting from social comparison.

Previous studies have explored the impact of self-construal on fear of negative evaluation and found that different types of self-construal can increase or decrease fear of negative evaluation (Levinson et al., 2011; Norasakkunkit & Kalick, 2002).

In China, Peng et al. (2019) found that fear of negative evaluation has a stronger predictive effect on social anxiety among individuals with an interdependent self-construal, whereas it has a weaker predictive effect among those with an independent self-construal. Ren et al. (2019) also found that interdependent self-construal is associated with higher levels of negative emotions such as anxiety and depression. Individuals with an interdependent self-construal exhibit stronger stress responses in acute psychological stress situations.

Individuals with independent self-construal focus more on personal efficacy rather than others’ opinions. They may view upward social comparison as irrelevant reference information, thereby reducing the impact of upward social comparison on fear of negative evaluation. In contrast, individuals with interdependent self-construal tended to define themselves through their relationships with others and were more sensitive to others’ evaluations. This sensitivity makes them more likely to interpret upward social comparisons as a threat to their social status, thereby intensifying fear of negative evaluation. Therefore, it is reasonable to propose Hypothesis 3:

**H3.**  *Self-construal moderates the relationship between upward social comparison and fear of negative evaluation. Specifically, upward social comparison has a stronger positive effect on fear of negative evaluation among individuals with interdependent self-construal, whereas the effect is weaker for those with independent self-construal.*

In addition, some studies have confirmed that the two self-construal types, as individual difference variables, can be temporarily and effectively activated not only in an experimental context (Brewer & Gardner, 1996) but also in various settings through instructional manipulations (Gardner et al., 1999; Oyserman & Lee, 2008). Therefore, self-construal is context-dependent. In this study, through the instructional priming of self-construal, we can better explore its moderating role in the link between upward social comparison and fear of negative evaluation.

**Figure 1:**
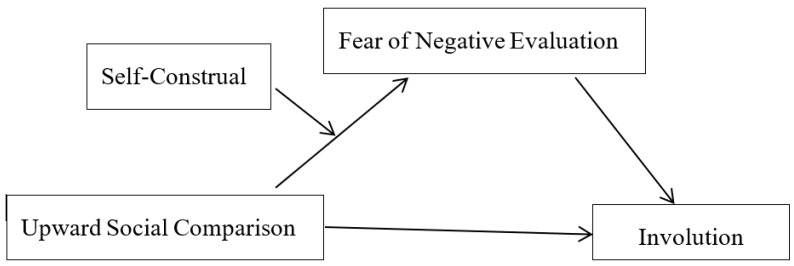
A moderated mediation model.

To sum up, the present research consists of two empirical studies designed to test the relationship between upward social comparison and involution, explore the mediating role of fear of negative evaluation in this link, and examine the moderating role of self-construal in the relationship between upward social comparison and fear of negative evaluation, with the aim of establishing a moderated mediation model (see Figure 1). It is hoped that this research can provide insights to help alleviate involution and promote the healthy development of Chinese college students.

## 3. Study 1

We aimed to explore the relationship between upward social comparison and involution as well as the mediating role of fear of negative evaluation in this link among Chinese college Students. Specifically, we examined the correlations among these variables and how upward social comparison and fear of negative evaluation predict involution.

### 3.1. Participants and Procedure

The sample in the current study consisted of 1549 Chinese college students, of which 51% were males (*n* = 795) and 49% were females (*n* = 754) (Mage = 20.11, SD = 0.96). Recruited on a voluntary basis from one university in Urumqi, the capital city of the Xinjiang Uyghur Autonomous Region of China, participants completed the following Chinese-version instruments and provided anonymous self-reports. They also reported demographic information, such as age and gender.

Teachers and graduates majoring in psychology at this university were invited to assist with the research process, including participant recruitment and scale administration. Each student received information regarding the purpose of this study, confidentiality protocols, and their right to withdraw from participation at any time. Informed consent was obtained from all participants, and strict confidentiality of their responses was ensured throughout the study.

### 3.2. Measures

#### 3.2.1. Upward Social Comparison

Based on the Upward Social Comparison Scale (Gibbons & Buunk, 1999), a standardized Chinese version, revised by Bai et al. (2013) was used in this study. It consists of 6 items on 5-point scales from 1 (strongly disagree) to 5 (strongly agree), (e.g., “I often compare myself with those who are better than me”), with high scores indicating a stronger tendency for upward social comparison (α = 0.88).

#### 3.2.2. Fear of Negative Evaluation

The Brief Fear of Negative Evaluation Scale (Leary, 1983) was used in this study. Z. Chen (2002) translated and validated the scale into standard Chinese. The scale consists of 12 items on 5-point scales from 1 (strongly disagree) to 5 (strongly agree), in which there are 4 reverse-scored items (e.g., “I am extremely afraid of others pointing out my shortcomings”), with high scores indicating greater fear of negative evaluation (α = 0.94).

#### 3.2.3. Involution

The Perceived Involution Questionnaire developed by Zhang et al. (2024) was used to measure involution. The scale consists of 18 items on 5-point scales from 1 (strongly disagree) to 5 (strongly agree) measuring four dimensions, namely Resource Scarcity (e.g., “The limited resources around me have an adverse effect on me”), Social Norms (e.g., “It is not enough to complete the minimum task in my study. Most people strive to do more”), Psychological Stress (e.g., “Studying makes me upset”), and Competitive Behavior (e.g., “People around me become outstanding through competition”) (αtotal = 0.96; α = 0.85, 0.83, 0.86, and 0.84, respectively), with higher scores indicating a greater level of involution.

### 3.3. Results

#### 3.3.1. Common Method Bias Test

The single-factor test was conducted to evaluate common method bias, a type of bias caused by the use of identical methods for data collection. According to an unrotated factor analysis of all items in the scales, the first factor accounted for 36.4%, which is lower than the 40% criterion (H. Zhou & Long, 2004), indicating that common method bias might not be an issue in this study.

#### 3.3.2. Descriptive Statistics and Correlation Analysis of Variables

The means, standard deviations, and correlations among all variables are presented in Table 1. Correlation analysis showed that upward social comparison was positively correlated with involution (r = 0.29, *p* < 0.01) and fear of negative evaluation (r = 0.29, *p* < 0.01). Fear of negative evaluation was positively correlated with involution (r = 0.27, *p* < 0.01). In addition, upward social comparison and fear of negative evaluation were positively correlated with all four dimensions of involution.

Although these results replicate much of the previous research, the most important consideration for this study was whether the evidence found here could support Hypothesis 2, which posits that fear of negative evaluation mediates the relationship between upward social comparison and involution. The criteria for the existence of a mediation effect include significant correlations among these three variables. As shown in Table 1, these conditions were all met, supporting the subsequent testing of the mediation model.

A mediation analysis (Wen & Ye, 2014) and model 4 in the Bootstrap method (Hayes, 2013) were used to test the model in which fear of negative evaluation mediated the relationship between upward social comparison and involution.

The mediation model was confirmed by confirmatory factor analysis, and goodness-of-fit indices were good, χ^2^/df = 14.87, CFI = 0.99, TLI = 0.99, RMSEA = 0.02, SRMR = 0.01.

As shown in Table 2, after controlling for the effects of gender and age, the results showed that upward social comparison was a significantly positive predictor of involution (β = 0.25, *p* < 0.001) and fear of negative evaluation (β = 0.13, *p* < 0.001).

When the mediating variable (fear of negative evaluation) was entered into the model, the predictive effect of upward social comparison on involution remained significant (β = 0.20, *p* < 0.001) and the predictive effect of fear of negative evaluation on involution was also significant (β = 0.41, *p* < 0.001). This indicated that there was a significant mediating effect of fear of negative evaluation between upward social comparison and involution (β = 0.05, *p* < 0.001).

The mediating effect was further tested using the Bootstrap method with 5000 resamples. The 95% confidence interval (CI) for the mediating effect was [0.04, 0.07], which did not contain zero, and the mediating effect accounted for 20.64% of the total effect. Collectively, these statistical results supported Hypothesis 2, which posits that fear of negative evaluation plays a significant mediating role in the relationship between upward social comparison and involution.

#### 3.3.3. Discussion

Consistent with our hypotheses, upward social comparison was positively correlated with involution and had a strong predictive effect on involution among Chinese college students. Specifically, when college students had a stronger sense of upward social comparison, they were more likely to engage in involutionary behaviors. Social comparison theory (Festinger, 1954) posits that individuals assess their self-worth by comparing themselves with others to fulfill self-evaluation needs. Upward social comparison, which involves comparing oneself with those who are better off, is a double-edged sword. On one hand, it reflects an agentic endeavor to achieve one’s competence and is conducive to the pursuit of efficacious goals. On the other hand, it represents vigilant efforts to avoid failure, which causes individuals to perceive deficits, experience a fear of negative evaluation, and ultimately engage in more intense anxiety and competitive behaviors.

These results provide initial support for the strong predictive effect of upward social comparison on involution and confirm a mediating role of fear of negative evaluation between upward social comparison and involution.

## 4. Study 2

After examining the predictive power of upward social comparison on involution and the mediating role of fear of negative evaluation between upward social comparison and involution, Study 2 aimed to validate the moderating role of self-construal between upward social comparison and fear of negative evaluation, thereby establishing a moderated mediation model. Specifically, we examined the correlations among upward social comparison, fear of negative evaluation, self-construal and involution. As two types of self-construal, both independent and interdependent self-construal were manipulated via instruction priming because it is easy to administer, its effectiveness has been widely validated, and it allows for a more direct understanding of the relationship between self and others.

Given that Chinese college students’ involution is motivated by upward social comparison and fear of negative evaluation, we investigated how self-construal moderates the link between upward social comparison and fear of negative evaluation, which reflects individuals’ distinct views of the self and expectations in social interactions.

### 4.1. Participants

The sample in the current study consisted of 392 Chinese college students, who were different from those in Study 1. They were recruited on a voluntary basis and randomly assigned to the independent self-construal group and the interdependent self-construal group, among whom 195 were males and 197 were females (Mage = 20.09, SD = 0.91). The independent self-construal group included 196 students (male = 97, female = 99), while the interdependent self-construal group also included 196 students (male = 98, female = 98).

Informed consent was obtained from all participants, and strict confidentiality of their responses was ensured throughout the study.

### 4.2. Measures

#### 4.2.1. Upward Social Comparison

Same as Study 1 (Bai et al., 2013; Gibbons & Buunk, 1999). It consists of 6 items on 5-point scales from 1 (strongly disagree) to 5 (strongly agree), (e.g., “I often compare myself with those who are better than me”), with high scores indicating a stronger tendency for upward social comparison (α = 0.92 in this study).

#### 4.2.2. Fear of Negative Evaluation

Same as Study 1 (Z. Chen, 2002; Leary, 1983). The scale consists of 12 items on 5-point scales from 1 (strongly disagree) to 5 (strongly agree), in which there are 4 reverse-scored items (e.g., “I am extremely afraid of others pointing out my shortcomings”), with high scores indicating greater fear of negative evaluation (α = 0.97 in this study).

#### 4.2.3. Involution

Same as Study 1 (Zhang et al., 2024). The scale consists of 18 items on 5-point scales from 1 (strongly disagree) to 5 (strongly agree) measuring four dimensions, namely Resource Scarcity (e.g., “The limited resources around me have an adverse effect on me”), Social Norms (e.g., “It is not enough to complete the minimum task in my study. Most people strive to do more”), Psychological Stress (e.g., “Studying makes me upset”), and Competitive Behavior (e.g., “People around me become outstanding through competition”) (αtotal = 0.93; α = 0.90, 0.90, 0.91, and 0.89, respectively in this study), with higher scores indicating a greater level of involution.

#### 4.2.4. Self-Construal

The Self-Construal Scale, designed by Singelis (1994), was used to assess individuals’ independent and interdependent views of the self. Y. Wang et al. (2008) translated and validated the scale into standard Chinese. This 24-item scale consists of two subscales, in which 12 items measure independent self-construal (e.g., “What I mainly care about is being able to take care of myself”) and 12 items measure interdependent self-construal (e.g., “Maintaining a harmonious relationship with others is very important to me”). Responses for both subscales are indicated on 7-point scales from 1 (strongly disagree) to 7 (strongly agree) in this study (α = 0.96 and α = 0.96 for the independence and interdependence subscales, respectively).

#### 4.2.5. Procedure

The instruction priming method was used in this study, which has been proven to be effective in the previous studies both in China (Yang et al., 2018) and internationally (Gardner et al., 1999; Neumann et al., 2009; Trafimow et al., 1991). Independent self-construal was activated with the following instruction: “Please spend 2 min listing three differences between yourself and your friends, as well as your expectations for yourself”. Interdependent self-construal was activated with the following instruction: “Please spend 2 min listing three commonalities between yourself and your friends, as well as their expectations for you”.

Self-construal scale was immediately administered to test the effectiveness of manipulation (Singelis, 1994). An independent samples *t*-test was conducted to compare the differences in self-construal measured in this study. A difference score was calculated by subtracting the total independent self-construal score from the total interdependent self-construal score. Higher difference scores indicated a stronger interdependent self-construal, while lower difference scores indicated a stronger independent self-construal. The results showed that in the independent self-construal group, scores on the independent self-construal items were significantly higher than those on the interdependent self-construal items, t = −5.22, *p* < 0.001. In the interdependent self-construal group, scores on the interdependent self-construal items were significantly higher than those on the independent self-construal items, t = 3.24, *p* < 0.001. These results indicated that the priming of self-construal was effective (see Table 3).

After completing this priming task, participants were asked to complete the Upward Social Comparison Scale, the Brief Fear of Negative Evaluation Scale, and the Perceived Involution Questionnaire.

### 4.3. Results

#### 4.3.1. Descriptive Statistics and Correlation Analysis of Variables

The means, standard deviations, and correlations among all variables are presented in Table 4. Upward social comparison was positively correlated with involution (r = 0.49, *p* < 0.01), positively correlated with fear of negative evaluation (r = 0.35, *p* < 0.01), as well as with both interdependent self-construal (r = 0.23, *p* < 0.01) and independent self-construal (r = 0.19, *p* < 0.01). Fear of negative evaluation was positively correlated with interdependent self-construal (r = 0.22, *p* < 0.01) and independent self-construal (r = 0.24, *p* < 0.01).

#### 4.3.2. Test of Fear of Negative Evaluation as a Mediator and Self-Construal as a Moderator

Following the steps for testing a moderated mediation model recommended by some researchers (Hayes, 2013; Wen & Ye, 2014), Study 2 mainly tested the mediating role of fear of negative evaluation between upward social comparison and involution, as well as the moderating role of self-construal between upward social comparison and fear of negative evaluation. Results indicated that there was an acceptable moderated mediation model, χ^2^/df = 3.81, CFI = 0.93, TLI = 0.90, RMSEA = 0.08, SRMR = 0.06. Model 7 of the PROCESS macro was conducted to verify the moderating mediation model (see Table 5). Upward social comparison positively predicted fear of negative evaluation (β = 0.32, *p* < 0.001). Self-construal also positively predicted fear of negative evaluation (β = 0.14, *p* < 0.001). Most importantly, the interaction between upward social comparison and self-construal significantly predicted fear of negative evaluation (β = 0.08, *p* < 0.05). These results indicated that self-construal moderated the relationship between upward social comparison and fear of negative evaluation, supporting Hypothesis 3.

**Figure 2:**
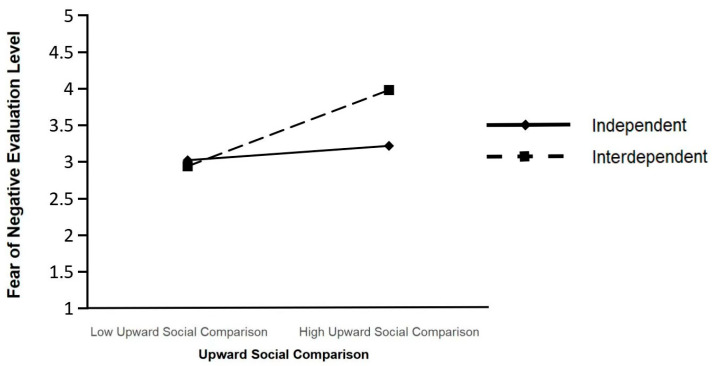
The moderating role of self-construal between upward social comparison and fear of negative evaluation.

Simple slope analysis was conducted by adding or subtracting one standard deviation from the mean of self-construal, aimed at clarifying the moderating effect of self-construal (see Figure 2). The results revealed that for individuals with a strong independent self-construal, upward social comparison had a weaker positive impact on fear of negative evaluation (β = 0.21, t = 3.02, *p* < 0.01). In contrast, for individuals with a strong interdependent self-construal, upward social comparison had a stronger positive impact on fear of negative evaluation (β = 0.43, t = 6.29, *p* < 0.001). These results further demonstrated that the association between upward social comparison and fear of negative evaluation was significantly moderated by self-construal: the positive association was stronger among individuals with the interdependent self-construal and weaker among those with the independent self-construal, thus providing direct support for Hypothesis 3.

### 4.4. Discussion

The present results showed that self-construal played a significant moderating role in the link between upward social comparison and fear of negative evaluation. To be specific, for people with the independent self-construal, upward social comparison had a weaker impact on fear of negative evaluation. For people with the interdependent self-construal, upward social comparison had a stronger impact on fear of negative evaluation.

According to the theory of self-construal (Markus & Kitayama, 1991), individuals with the independent self-construal perceive the self as a bounded, unique, and integrated entity—a dynamic center of awareness, emotion, judgment, and action that is organized into a distinctive whole and set in contrast to other individuals and the social context. They emphasize personal autonomy, internal attributes, and separateness from others.

In contrast, individuals with the interdependent self-construal view the self as fundamentally connected to others and embedded within social relationships. They regard the self as interdependent with the surrounding context, where their behaviors are largely determined and organized by the perceived thoughts, feelings, and actions of relevant others. Relatedness, harmony, and the “self-in-relation-to-other” are the distinctive features of interdependent self-construal.

Obviously, self-construal is sustained by an adaptive attribute that is attracted to the variety and diversity of personal experiences and a cognitive style that attends to self-views. From the present study, it is clear that independent self-construal might be conducive to eliciting a defensive reaction against the negative impact of upward social comparison on fear of negative evaluation.

## 5. General Discussion

The present research investigated the relationship between upward social comparison and involution, the mediating role of fear of negative evaluation in this link and the moderating role of self-construal in the association between upward social comparison and fear of negative evaluation among Chinese college students, with the aim of establishing a moderated mediation model of these variables.

Based on the proposition suggested by some Chinese researchers (Zhang et al., 2024) that a high level of upward social comparison makes a positive contribution to involution, the hypothesis that upward social comparison is significantly related to involution of Chinese college students was supported by the present findings.

As social comparison theory (Festinger, 1954) indicates, people often evaluate their own abilities and values by comparing themselves with more outstanding individuals. The perceived gap may elicit self-evaluation threats, which in turn lead to increased effort to alleviate anxiety. However, such efforts often fail to yield equivalent returns, creating an imbalance between input and output and ultimately resulting in involution (Yang et al., 2022). In a country with increasingly fierce competition like China, upward social comparison seems to have become a widespread standard or norm (Tao & Xu, 2014). When competitive values prompt college students to reach a consensus that “the harder you study, the safer you are”, involution is naturally strengthened through multiple aspects such as cognition, emotion, and behavior. Consistent with previous studies conducted in China (Lin, 2021; J. Wang, 2021), the present study provides evidence that upward social comparison can positively predict involution.

Second, this research tested whether the link between upward social comparison and involution was mediated by fear of negative evaluation. The present results confirm the mediating role among Chinese college students. That is, upward social comparison predicts involution via fear of negative evaluation. Specifically, the higher the level of upward social comparison, the greater the fear of negative evaluation and, consequently, a higher level of involution.

Some researchers have suggested that there is a relationship between upward social comparison, fear of negative evaluation, and involution in which strong upward social comparison is linked to higher fear of negative evaluation, which in turn is positively associated with involution both in China (Fan & Zhang, 2024) and internationally (Adelman & Dasgupta, 2018; Weeks & Howell, 2012). Lending support to social comparison theory (Festinger, 1954), the present research indicates that upward social comparison significantly increases the level of fear of negative evaluation by eliciting individuals’ doubts about their abilities and anxieties about their social image. To alleviate this sense of fear and maintain their social status, individuals make great efforts to compete for resources and comply with norms, which eventually results in involution. In increasingly competitive Chinese universities, there is a growing emphasis on grades and academic performance. This drives Chinese college students to engage in upward social comparison to avoid negative evaluations, thereby systematically intensifying involution (Qin & Dai, 2022; T. Zhou & Miao, 2023).

Consistent with such theorizing, the present findings suggest that upward social comparison is associated with fear of negative evaluation, which in turn is correlated with increased involution. For Chinese college students who engage in upward social comparison, fear of negative evaluation is not only a direct psychological outcome caused by upward social comparison, but also a core mechanism through which involution is reinforced. In the present research, it seems that a positive social comparison appears to be particularly important to the development of Chinese college students.

Finally, this research tested whether the link between upward social comparison and fear of negative evaluation was moderated by self-construal. The current results confirm Hypothesis 3 and indicate the moderating effect for Chinese college students. Specifically, for those with the independent self-construal, upward social comparison has a weaker impact on fear of negative evaluation. In contrast, for those with the interdependent self-construal, upward social comparison has a stronger impact on fear of negative evaluation. Nisbett et al. (2001) affirmed that there are some fundamental differences in information processing between independent self-construal and interdependent self-construal. Individuals with the independent self-construal emphasized their own abilities and uniqueness while individuals with the interdependent self-construal focused on group relationships. This difference leads to varying levels of fear of negative evaluation caused by upward social comparison.

Individuals with the independent self-construal have a lower sensitivity to external evaluations and are more likely to view upward social comparison as opportunities for self-improvement mainly because their self-worth stems from internal beliefs. In contrast, individuals with the interdependent self-construal have a higher sensitivity to external evaluations and are more likely to view upward social comparison as threats to their development mainly because their self-worth depends on external recognition. Consistent with the conclusion of Tian et al. (2023), the present study provides a new perspective for understanding the role of self-construal in moderating the relationship between upward social comparison and fear of negative evaluation. Through the instructional priming of self-construal, we can better understand the strength and direction of self-construal’s role in the link between upward social comparison and fear of negative evaluation.

In summary, in the course of examining the relationship between upward social comparison, fear of negative evaluation, self-construal and involution, both upward social comparison and fear of negative evaluation are found to be important indexes to better interpret involution of Chinese college students. These two variables might be fundamental for involution and can be regarded as the precursors to the involution of Chinese college students. Furthermore, the present research demonstrates the complex interplay between upward social comparison, fear of negative evaluation and self-construal—an interplay that some researchers suggested an exploration (Zhang et al., 2024). Zhang et al. (2024) argued that such concepts as upward social comparison, fear of negative evaluation and involution that had inspired some separate studies and were often explored as separate entities, were in fact inextricably correlated and needed to be examined together. The present research has explored these concepts simultaneously and has empirically demonstrated how upward social comparison, fear of negative evaluation and self-construal might be related to each other and contribute to involution among Chinese college students. Consistent with theorizing originating in social comparison theory (Festinger, 1954) and self-construal theory (Markus & Kitayama, 1991), upward social comparison, fear of negative evaluation and self-construal have been found to be strongly associated. This finding aligns with results from a large body of research on young people across China and supports the prominent generalization that cultural values exert a significant influence on young people’s development.

## 6. Research Limitations and Future Directions

Although the main advantage of the study lies in its ability to test the relationship between upward social comparison and involution, verify the mechanism underlying this link, and further validate the moderating role of self-construal in the relationship between upward social comparison and fear of negative evaluation, it is not without limitations.

First, not all factors that might be significant in upward social comparison and involution relationships were included in the present study. It will be important in future studies to incorporate other sources, such as personality traits, motivation and socioeconomic status that might potentially mediate the relationship between upward social comparison and involution. Researchers should further explore the joint or differential roles of these factors in the link between upward social comparison and involution in greater detail.

Second, although the relationship between upward social comparison and involution is important, the cross-sectional data used in the present research limit the possibility of drawing causal inferences. Therefore, future research should adopt a longitudinal design combined with scales, experiments, and interviews to better comprehend the development and changes in Chinese college students’ involution and verify the causal relationships among these variables. Through these efforts, a more comprehensive understanding of Chinese college students’ involution can be achieved, and more effective solutions can be provided to promote the healthy development of Chinese young people.

## 7. Conclusions

In a society or an environment with limited resources, individuals keep increasing their efforts. However, no overall progress is achieved, resulting only in involution. Based on two studies, the present research explores the relationship among involution, upward social comparison, fear of negative evaluation and self-construal, and aims to construct a moderated mediation model. The results support this model, showing that upward social comparison indirectly contributes to involution by exacerbating fear of negative evaluation, and this mediating pathway is significantly moderated by an individual’s type of self-construal.

We argue that self-construal is an individual-difference construct with some unique features and strengths. Specifically, the independent self-construal reflects autonomy-focused endeavors that derive objective attitudes from uniqueness, endorse self-worth, and direct self-actualization behaviors to maximize beneficial outcomes in social interactions. In contrast, the interdependent self-construal represents relationship-focused mechanisms that derive emotions from social contact, take others’ perspectives, and exhibit norm-consistent behaviors to minimize adverse consequences in social interactions.

We found that the independent self-construal buffers the effect of upward social comparison on fear of negative evaluation, whereas the interdependent self-construal strengthens this effect. Therefore, self-construal, as a cognitive structure reflecting individual differences, helps elucidate the psychological processes underlying the link between upward social comparison and fear of negative evaluation.

In conclusion, this research enriches and extends the existing literature on the factors influencing involution. Most previous studies have examined the relationship between fear of negative evaluation and negative emotions. In contrast, the present research introduces fear of negative evaluation into the context of involution, revealing its positive predictive effect on involution. Moreover, it demonstrates that involution driven by upward social comparison largely stems from individuals’ fear of negative evaluation. By establishing the relationship among upward social comparison, fear of negative evaluation, and involution, this research validates the mediating role of fear of negative evaluation. This mediating pathway elucidates the mechanism through which exposure to others’ greater effort drives individuals into involution, with fear of negative evaluation serving as the underlying driving force.

Furthermore, by examining the moderating role of self-construal in the link between upward social comparison and fear of negative evaluation, this research verifies that independent self-construal alleviates the adverse effect of upward social comparison on fear of negative evaluation. Individuals with an interdependent self-construal root their self-concept in social relationships, emphasizing connection and harmony with others. This makes them particularly sensitive to evaluations or feedback derived from social comparison. By contrast, those with an independent self-construal focus on their autonomy and uniqueness, rely less on others’ judgments, and are thus relatively insensitive to information from social comparison. The present study demonstrates that in the Chinese cultural context characterized by an emphasis on social connectedness and social recognition, independent self-construal exerts a positive effect on mitigating fear of negative evaluation caused by upward social comparison. By integrating these four variables, this research constructs a moderated mediation model, providing a more comprehensive and in-depth theoretical framework for future research.

In addition, the present research also has practical implications for the development of Chinese college students. First, diverse evaluation criteria should be established so that these young adults can recognize their performance in different domains and thereby fundamentally reduce the impact of upward social comparison on involution. External evaluations should be weakened to enable them to respond appropriately to upward social comparison and thus alleviate fear of negative evaluation. Second, the proactive and protective role of the independent self-construal highlights the importance of cultivating a positive self-worth system. Independent self-construal should be fostered among Chinese college students to develop internal motivation and personalized goals. As a result, these young adults can more effectively transform the pressure from upward social comparison into intrinsic motivation for their own growth and progress.

## Figures and Tables

**Table 1:** Means, Standard Deviations, and Correlations Between Variables (*n* = 1549).

Variable	*M*	*SD*	1	2	3	4	5	6	7
Upward social comparison	3.80	0.94	-						
Fear of negative evaluation	3.22	0.41	0.29 **	-					
Involution	3.62	0.83	0.29 **	0.27 **	-				
Psychological stress	3.82	0.93	0.27 **	0.27 **	0.27 **	-			
Social norms	3.84	0.95	0.27 **	0.25 **	0.94 **	0.83 **	-		
Competitive behavior	3.85	0.93	0.27 **	0.24 **	0.95 **	0.85 **	0.84 **	-	
Resource scarcity	3.83	0.97	0.27 **	0.26 **	0.92 **	0.84 **	0.83 **	0.84 **	-

Note. ** *p* < 0.01.

**Table 2:** Regression Analysis of Mediating Results in the Relationship between Upward Social Compassion and Fear of Negative Evaluation.

Step and Variable	Involution (Step 1)		Fear of Negative Evaluation (Step 2)		Involution (Step 3)	
	β	*t*	β	*t*	β	*t*
Gender (male = 1, female = 2)	0.01	0.23	−0.02	−0.87	0.002	0.05
age	0.01	0.64	0.02	0.09	0.02	0.97
Upward social comparison	0.25 ***	11.77	0.13 ***	11.89	0.20 ***	9.14
Fear of negative evaluation					0.41 ***	8.17
R^2^	0.08		0.09		0.12	
F	46.47 ***		48.20 ***		53.02 ***	

Note. *** *p* < 0.001.

**Table 3:** Effectiveness Test of Self-Construal Priming (M ± SD).

	Interdependent Self-Construal	Independent Self-Construal	*t*	Cohen’s *d*
Interdependent Self-Construal Group	64.41 ± 13.78	58.16 ± 14.84	3.24 ***	0.32
Independent Self-Construal Group	59.61 ± 15.80	65.86 ± 14.68	−5.22 ***	0.52

Note. *** *p* < 0.001.

**Table 4:** Means, Standard Deviations, and Correlations Between Variables (*n* = 392).

Variable	*M*	*SD*	1	2	3	4	5	6	7	8	9
Upward social comparison	3.52	0.94	-								
Fear of negative evaluation	3.29	0.97	0.35 **	-							
Interdependent self-construal	5.15	0.62	0.23 **	0.22 **	-						
Independent self-construal	5.17	0.63	0.19 **	0.24 **	0.87 **	-					
Involution	3.51	0.74	0.49 **	0.53 **	0.30 **	0.30 **	-				
Psychological stress	3.69	0.90	0.46 **	0.38 **	0.28 **	0.18 **	0.75 **	-			
Social norms	3.47	0.99	0.31 **	0.43 **	0.23 **	0.25 **	0.82 **	0.52 **	-		
Competitive behavior	3.45	0.93	0.37 **	0.43 **	0.20 **	0.24 **	0.79 **	0.43 **	0.56 **	-	
Resource scarcity	3.43	0.96	0.40 **	0.41 **	0.23 **	0.26 **	0.77 **	0.44 **	0.48 **	0.50 **	-

Note. ** *p* < 0.01.

**Table 5:** Test of the Moderated Mediation Model.

Independent Variable	Fear of Negative Evaluation		Involution	
	β	*t*	β	*t*
Gender (male = 1, female = 2)	−0.02	−0.17	0.11	1.79
age	−0.01	−0.02	−0.02	−0.27
Upward social comparison	0.32	6.50 ***	0.28	8.38 ***
×Self-construal	0.14	3.79 ***		
Upward social comparison ×self-construal	0.08	1.96		
Fear of negative evaluation			0.31	9.81 ***
R	0.39		0.63	
R^2^	0.15		0.39	
F	14.49 ***		63.65 ***	

Note. *** *p* < 0.001.

## Data Availability

The data that support the findings of this research are available from the corresponding author upon reasonable request.
